# The VEGF rise in blood of bevacizumab patients is not based on tumor escape but a host-blockade of VEGF clearance

**DOI:** 10.18632/oncotarget.11084

**Published:** 2016-08-05

**Authors:** Lejla Alidzanovic, Patrick Starlinger, Dominic Schauer, Thomas Maier, Alexandra Feldman, Elisabeth Buchberger, Judith Stift, Ulrike Koeck, Lorand Pop, Birgit Gruenberger, Thomas Gruenberger, Christine Brostjan

**Affiliations:** ^1^ Department of Surgery, Medical University of Vienna, General Hospital, 1090 Vienna, Austria; ^2^ Department of Pathology, Medical University of Vienna, General Hospital, 1090 Vienna, Austria; ^3^ Department of Neuroimmunology, Medical University of Vienna, Center for Brain Research, 1090 Vienna, Austria; ^4^ Department of Internal Medicine, Hospital of The Merciful Brothers, 1020 Vienna, Austria; ^5^ Current address: Department of Surgery I, Rudolf Foundation Clinic, 1030 Vienna, Austria

**Keywords:** bevacizumab, colorectal carcinoma, liver metastasis, plasma, vascular endothelial growth factor

## Abstract

Vascular endothelial growth factor (VEGF) has become a major target in cancer treatment as it promotes tumor angiogenesis. Therapy with anti-VEGF antibody bevacizumab reportedly induces high levels of circulating VEGF which may potentially contribute to resistance. Based on animal or computational models, mechanisms of VEGF induction by bevacizumab have been proposed but not verified in the clinical setting. Hence, we evaluated sixty patients with colorectal cancer metastases for changes in plasma VEGF during neoadjuvant/conversion and adjuvant chemotherapy with or without bevacizumab. VEGF expression was assessed in tissue sections of liver metastases. The VEGF source was investigated with *in vitro* cultures of tumor, endothelial cells, fibroblasts and platelets, and potential protein stabilization due to anti-VEGF therapy was addressed. A VEGF rise was observed in blood of bevacizumab patients but not in chemotherapy controls, and VEGF was found to be largely complexed by the antibody. A comparable VEGF increase occurred in the presence (neoadjuvant) and absence of the tumor (adjuvant). Accordingly, VEGF expression in tumor tissue was not determined by bevacizumab treatment. Investigations with isolated cell types did not reveal VEGF production in response to bevacizumab. However, antibody addition to endothelial cultures led to a dose-dependent blockade of VEGF internalization and hence stabilized VEGF in the supernatant. In conclusion, the VEGF rise in cancer patients treated with bevacizumab is not originating from the tumor. The accumulation of primarily host-derived VEGF in circulation can be explained by antibody interference with receptor-mediated endocytosis and protein degradation. Thus, the VEGF increase in response to bevacizumab therapy should not be regarded as a tumor escape mechanism.

## INTRODUCTION

Angiogenesis is the process of capillary sprouting from pre-existing vessels and is essential to many physiological and pathological conditions [1]. The role of angiogenesis in supporting tumor growth and metastasis has been a major focus of cancer research over the past decades and vascular endothelial growth factor (VEGF) has been identified as one of the central mediators in this process [2]. There are several members of the VEGF family, including VEGF-A, B, C, D, E and placenta growth factor (PlGF) [3]. VEGF-A (generally called VEGF) is of particular importance in tumor angiogenesis. It is secreted by cancer cells [4] and various other cell types, including stromal fibroblasts [5], endothelial cells [6] and platelets [7]. Based on alternative splicing, different VEGF isoforms are created which, depending on the presence of the heparin binding domain, are known as primarily soluble and diffusible isoforms (VEGF-121), matrix-bound isoforms (VEGF-189, VEGF-206) or variants with mixed properties (VEGF-165) [8]. VEGF binds to specific high-affinity receptor tyrosine kinases [2] with three major types of VEGF receptors: VEGFR-1 (Flt-1), VEGFR-2 (KDR, Flk-1) and VEGFR-3 (Flt-4). VEGF-A binds to VEGFR-1 and VEGFR-2, which are predominantly expressed by endothelial cells while VEGFR-3 is mostly found on lymphatic vessels and interacts with VEGF-C and VEGF-D. By binding to its receptors, VEGF induces endothelial cell mitosis, migration, survival, and vascular permeability [2].

The overexpression of VEGF in the tumor environment leads to the development of blood vessels with structural abnormalities and functional defects, which support tumor expansion and dissemination [9, 10]. Numerous studies showed that increased levels of VEGF correlate with a higher tumor grade, incidence of metastasis and with a poor clinical outcome of cancer patients [4, 11]. In the attempt to block tumor angiogenesis, the VEGF pathway has become a prime target in cancer treatment. The first anti-VEGF drug approved for clinical application was bevacizumab (Avastin®, Roche), a humanized monoclonal antibody directed against all VEGF-A isoforms, which has shown clinical benefit and efficacy in several types of malignancies including metastatic colorectal cancer (mCRC). Several clinical trials have reported a significantly increased response rate and improved overall and progression-free survival of patients with mCRC receiving bevacizumab in addition to standard chemotherapy [12–15].

Paradoxically, intravenous administration of bevacizumab leads to an increase of VEGF blood levels in patients [16], which is considered to be a feedback mechanism and a potential pharmacodynamic marker of VEGF inactivation [17]. However, most of the circulating VEGF is antibody-bound and hence inactive [18, 19]. Of interest, a rise in VEGF blood concentration has not only been reported for bevacizumab therapy but has also been observed for other VEGF-targeted approaches such as anti-VEGFR-2 antibodies [17, 20], soluble VEGF receptor competitors [21] or tyrosine kinase inhibitors of VEGF receptor activity [22]. The response is generally rapid (within hours) and reaches almost maximal levels within 1-3 days after drug administration [17, 23].

The source of this “VEGF feedback induction” has not been conclusively revealed to date. Animal studies suggested that the injection of bevacizumab leads to a more hypoxic tumor environment, which activates a rescue pathway via hypoxia inducible factor 1α, leading to the increased expression of VEGF [24–26]. However, the analysis of VEGF in normal and tumor-bearing mice that received VEGF receptor-targeted therapy showed a rapid VEGF increase in both groups, suggesting that the VEGF feedback might be host–derived [17, 21]. With respect to the potential source of host VEGF induction, animal studies as well as computational models have produced a variety of explanations such as increased VEGF expression by hepatocytes [21] or other mouse tissues [17], decreased clearance from circulation [27] or re-localization from tissue to circulation [28].

With respect to patient therapy, the issue of VEGF induction upon anti-VEGF treatment is of central importance as it might represent a tumor escape mechanism which requires counter-action. In order to elucidate the regulation of VEGF induction by bevacizumab therapy in a clinical setting, we thus investigated sixty patients with colorectal liver metastases who received chemotherapy with or without the addition of bevacizumab. We determined changes of circulating VEGF levels during neoadjuvant and adjuvant treatment. Furthermore, by *in vitro* analyses with human cell cultures and tissues, we addressed the mechanism and source of VEGF accumulation in response to bevacizumab therapy.

## RESULTS

Among the patients who were enrolled in our study and received neoadjuvant (or conversion) treatment with chemotherapy, forty-five were treated with bevacizumab and fifteen without. The analysis of the patient collective showed no significant difference between the two treatment arms with respect to age, sex, number of treatment cycles, response to therapy, localization of the primary tumor and the extent of surgery (Table 1). While the majority of patients had the primary tumor resected prior to study inclusion, twelve patients were treated in a synchronous setting with resection of both, primary and liver metastases. With respect to the neoadjuvant/conversion collective, surgery could not be performed on thirteen patients. A total of thirty-two patients were also analyzed in the adjuvant setting, twenty-six with and six without bevacizumab treatment. No significant difference was found between these two groups with respect to age, sex, localization of the primary tumor and response to neoadjuvant therapy (Table 2).

**Table 1:** Demographics and clinical characteristics of mCRC patients investigated during neoadjuvant treatment

Parameter	Chemo (N=15)	Beva (N=45)	p-value
**Age, years**			
Median (range)	66.8 (54.9 – 79.8)	65.8 (39.7 – 80.5)	0.330
**Sex, N (%)**			
Male	9 (60%)	27 (60%)	1.000
Female	6 (40%)	18 (40%)	
**Primary, N (%)**			
Colon	7 (46.7%)	28 (62.2%)	0.290
Rectum	8 (53.3%)	17 (37.8%)	
**Resectability, N (%)**			
Initially resectable	13 (86.7%)	29 (64.4%)	0.192
Initially unresectable	2 (13.3%)	16 (35.6%)	
**Surgery, N (%)**			
Synchronous	2 (13.3%)	10 (22.2%)	0.395
Metachronous	11 (73.3%)	24 (53.3%)	
No liver resection	2 (13.3%)	11 (24.4%)	
**Chemo cycles, N**			
Median (range)	5 (3-12)	6 (3-19)	0.415
**Beva cycles, N**			
Median (range)		5 (3-19)	
**RECIST, N (%)**			
Partial response	12 (80%)	25 (55.6%)	0.221
Stable disease	1 (6.7%)	10 (22.2%)	
Progressive disease	2 (13.3%)	10 (22.2%)	
**Response, N (%)**			
Responder (CR+PR)	12 (80%)	25 (55.6%)	
Non-responder (SD+PD)	3 (20%)	20 (44.4%)	0.092

Abbreviations: Beva, patients treated with chemotherapy and bevacizumab; Chemo, patients treated with chemotherapy only; N, number.

**Table 2:** Demographics and clinical characteristics of mCRC patients investigated during adjuvant treatment

Parameter	Chemo (N=6)	Beva (N=26)	p-value
**Age, years**			
Median (range)	69.0 (54.9-77.9)	63.7 (41.8-77.5)	0.334
**Sex, N (%)**			
Male	5 (83.3%)	16 (61.5%)	
Female	1 (16.7%)	10 (38.5%)	0.637
**Primary, N (%)**			
Colon	3 (50%)	15 (57.7%)	1.000
Rectum	3 (50%)	11 (42.3%)	
**Surgery, N (%)**			
Synchronous	1 (16.7%)	9 (34.6%)	0.637
Metachronous	5 (83.3%)	17 (65.4%)	
**Neo chemo cycles, N**			
Median (range)	5.5 (4-8)	6 (4-10)	0.430
**Neo beva cycles, N**			
Median (range)		5 (3-9)	
**Neo RECIST, N (%)**			
Partial response	6 (100%)	19 (73.1%)	
Stable disease	0 (0%)	5 (19.2%)	
Progressive disease	0 (0%)	2 (7.7%)	0.356
**Neo response, N (%)**			
Responder (CR+PR)	6 (100%)	19 (73.1%)	
Non-responder (SD+PD)	0 (0%)	7 (26.9%)	0.296
**Adj chemo cycles, N**			
Median (range)	6 (3-6)	6 (4-8)	0.218
**Adj beva cycles, N**			
Median (range)		6 (4-8)	

Abbreviations: Adj, adjuvant; Beva, patients treated with chemotherapy and bevacizumab; Chemo, patients treated with chemotherapy only; Neo, neoadjuvant; N, number.

### Plasma VEGF increases after neoadjuvant as well as adjuvant treatment with bevacizumab but is bound by the neutralizing antibody

**Figure 1:**
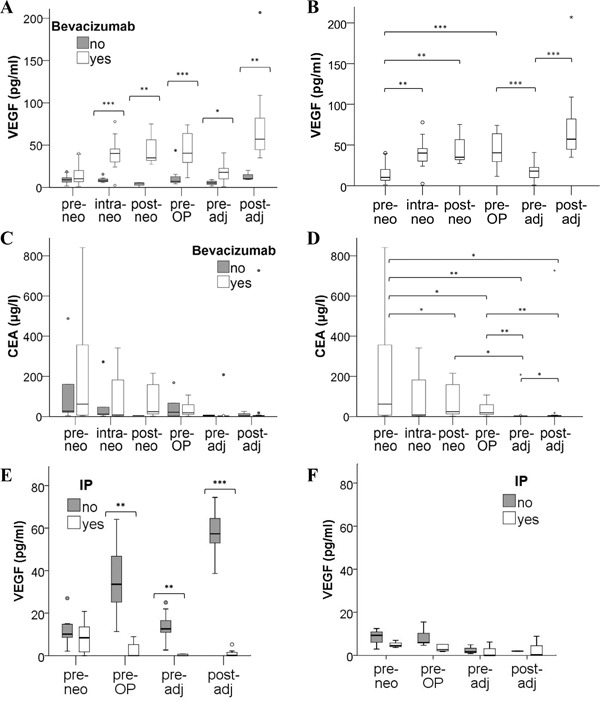
Distribution of VEGF plasma levels in mCRC patients under therapy Blood samples from mCRC patients treated with chemotherapy with or without the addition of bevacizumab were collected before, during and after neoadjuvant treatment (pre-, intra-, post-neo), immediately before surgery (pre-OP) and before as well as after adjuvant therapy (pre-, post-adj). Circulating VEGF was determined in plasma **A, B.** and tumor marker CEA was measured in serum **C, D.** Statistical differences between treatment arms are indicated in A and C, while significant changes during therapy of the bevacizumab group are illustrated in B and D. Please note that extreme values and statistical outliers have been omitted from graphs C and D to improve boxplot resolution. **E, F.** To differentiate between free and antibody-neutralized VEGF molecules, plasma samples of (E) 13 bevacizumab patients and of (F) 5 chemotherapy controls were subjected to an immunoprecipitation (IP) procedure to remove all antibody-bound VEGF prior to the detection of remaining, free VEGF by ELISA. (* p<0.05; ** p<0.01; *** p<0.001).

The levels of VEGF were significantly increased (baseline median value of 10 pg/ml VEGF raised to 40 pg/ml within 2 cycles; p < 0.001) in the plasma of patients receiving neoadjuvant treatment with bevacizumab (Figure 1A and 1B) while no VEGF rise was observed for patients receiving chemotherapy without anti-VEGF antibody. At the beginning of adjuvant therapy, VEGF values had returned to almost baseline (median 18 pg/ml VEGF), and a comparable increase of VEGF levels was then observed during adjuvant treatment in the bevacizumab group (median 57 pg/ml VEGF; p < 0.001) but not in the chemotherapy control collective.

To address the pertinent question whether plasma VEGF was antibody-bound and hence inactive (despite the elevated levels) in the bevacizumab-treated patients, immunoprecipitation experiments were conducted. When immunoglobulin (including the bevacizumab antibody) was removed from plasma samples, levels of unbound (free) VEGF were hardly detectable in the entire neoadjuvant and adjuvant treatment period of bevacizumab patients (Figure 1E). In contrast, the immunoprecipitation procedure did not significantly alter the VEGF levels detected at baseline (prior to bevacizumab administration) or in plasma samples of chemotherapy controls (Figure 1F).

The analysis of the tumor marker CEA revealed a decrease after neoadjuvant therapy (Figure 1C and 1D) and the levels remained within a normal range after surgery and during the adjuvant treatment period, without significant difference between the two treatment arms. Our results further showed that a decrease in CEA levels correlated with response to neoadjuvant treatment based on RECIST evaluation (p = 0.009; Supplementary Figure S1), while there was no correlation of VEGF levels with response to therapy (data not shown).

### VEGF expression in resected CRC liver metastases is not determined by neoadjuvant bevacizumab therapy

**Figure 2:**
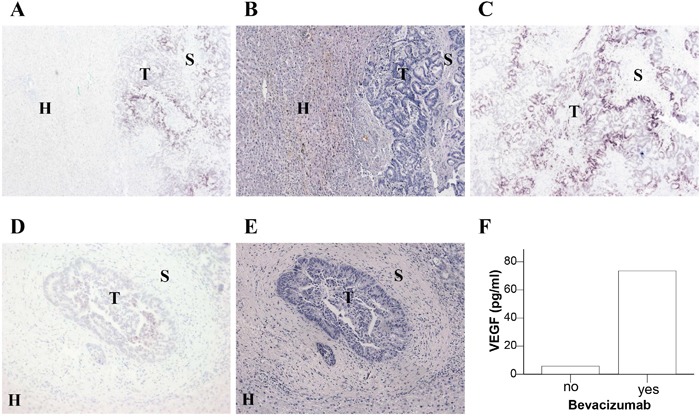
Expression of VEGF mRNA in liver sections of CRC metastases Resected liver metastases from two CRC patients who were neoadjuvantly treated without bevacizumab **A-C.** or with bevacizumab **D, E.** were analyzed for VEGF mRNA expression by *in situ* hybridization (A, C, D). Comparable sections with hematoxylin and eosin staining (B, E) are shown. The location of tumor cells (T), stromal cells (S) and hepatocytes (H) is indicated. **F.** Plasma VEGF levels of these two patients at the time of surgery.

Despite the observation that the “VEGF feedback induction” was also observed during adjuvant treatment i.e. in the absence of tumor, we investigated VEGF expression in resected liver metastases of 6 patients after neoadjuvant therapy. Apart from the cancer cells and tumor stroma, we questioned whether hepatocytes might function as VEGF source as previously proposed for mice under VEGF receptor blockade [21]. VEGF expression was detected at the mRNA level by *in situ* hybridization (ISH) but not at the protein level due to a low detection limit of VEGF by immunohistochemical staining. The analysis showed that VEGF levels detected in plasma did not correlate with VEGF expression in resected CRC liver metastases (Figure 2 and Table 3). The expression of VEGF in the tumor cells was not determined by neoadjuvant treatment with or without bevacizumab. Furthermore, there was no detectable expression of VEGF in the adjacent liver tissue.

**Table 3:** Expression of VEGF mRNA in tumor, stroma and hepatocytes of resected liver metastases of CRC patients as detected by *in situ* hybridization

Patient	Therapy	Pre-OP plasma VEGF (pg/ml)	VEGF mRNA in tumor	VEGF mRNA in stroma	VEGF mRNA in hepatocytes
1	Chemo	5.75	+++	-	-
2	Chemo	6.01	-	-	-
3	Chemo	5.59	-	-	-
4	Beva	72.72	-	-	-
5	Beva	71.88	++	-	-
6	Beva	73.51	+	-	-

Abbreviations: Beva, patients treated neoadjuvantly with chemotherapy and bevacizumab; Chemo, patients treated with chemotherapy only; Pre-OP, pre-operative.

### No alteration in VEGF content of platelets by bevacizumab therapy

**Figure 3:**
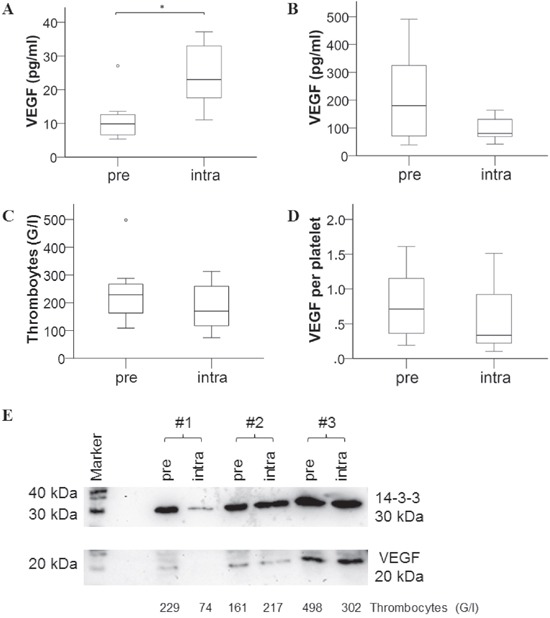
Assessment of VEGF content in platelets of mCRC patients before and after bevacizumab therapy Blood samples were retrieved from 7 patients before (pre) and after 1-2 weeks (intra) of chemotherapy with bevacizumab treatment and analyzed for **A.** VEGF plasma levels, **B.** VEGF levels in serum, **C.** platelet count; **D.** VEGF content per platelet was then calculated by subtraction of plasma from serum values and adjusted to platelet count. **E.** VEGF as detected by immunoblotting in protein extracts of isolated platelets is shown for 3 of 7 investigated patients (compared to 14-3-3 “housekeeper” protein for loading control and platelet blood count of the respective sample).

To identify a possible host-derived source for VEGF in response to bevacizumab, we first investigated platelets, since they are known to contain and release large amounts of VEGF upon activation [7]. Hence, seven mCRC patients were analyzed at baseline and 1-2 weeks after therapy with bevacizumab. The VEGF content of platelets was determined based on VEGF measurements in plasma (Figure 3A) and serum (Figure 3B), i.e. plasma values (representing freely circulating VEGF) were subtracted from serum values (containing additional VEGF released upon platelet activation during blood coagulation) and divided by platelet count (Figure 3C). Despite a pronounced VEGF increase in plasma, there was no significant difference in platelet VEGF content before and after bevacizumab therapy (Figure 3D). Also, there was no difference in platelet count and serum VEGF levels. To substantiate our finding we further isolated platelets and compared VEGF in platelet extracts by immunoblotting (Figure 3E). Accordingly, the analyses revealed that bevacizumab therapy had no impact on the platelet VEGF content.

### Bevacizumab has no influence on cellular expression of VEGF in tumor and stromal cells

**Figure 4:**
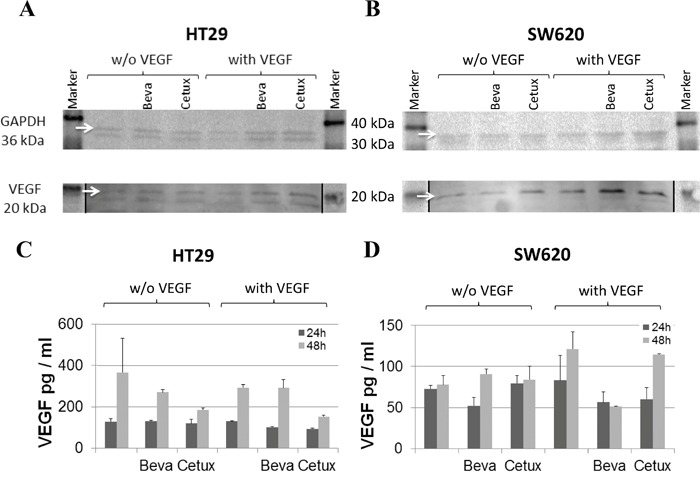
VEGF expression in colorectal cancer cell lines in response to bevacizumab treatment Protein extracts were prepared from HT29 **A, C.** or SW620 **B, D.** cells after pre-conditioning with or without 100 pg/ml hrVEGF for 24 h and subsequent incubation without or with 50 μg/ml bevacizumab or cetuximab, for negative control. Cell extracts were evaluated for VEGF content by immunoblotting (A, B) after 24 h or by ELISA (C, D) after 24 h and 48 h of antibody exposure. Images of immunoblots have been processed with Adobe Photoshop CS6 software to adjust brightness and contrast (autocontrast) of the entire image; stiched image parts are separated by a black line.

In addition to cancer cells, tumor stroma is known as a source of host-derived VEGF supporting tumor expansion [4–6]. Therefore, we proceeded to investigate the influence of bevacizumab on the cellular expression of VEGF by separate *in vitro* cell cultures. The two CRC cell lines HT29 and SW620 harbor mutations in the K-ras and p53 genes which are associated with a strong upregulation of VEGF expression [29, 30]. Hence, these cells showed high levels of VEGF release which was not further increased when exposed to hypoxia (data not shown). In addition to the two CRC cell lines, primary human fibroblasts and endothelial cells were analyzed. Cell cultures were either left untreated or exposed to human recombinant VEGF-165 (hrVEGF) for 24 h prior to treatment with bevacizumab or cetuximab, for negative control. Immunoblotting of cell extracts prepared from colorectal cancer cells (in 2 independent experiments) showed no enhancement of VEGF expression after incubation with bevacizumab for 24 h (Figure 4A and 4B). Comparable results were seen after 48 h (data not shown) or when intracellular VEGF levels were measured by ELISA (Figure 4C and 4D)

When primary human fibroblasts (N=2) and ECs (N=4 experiments) were investigated, VEGF protein expression was undetectable in extracts of these stromal cells and exposure to bevacizumab did not induce VEGF protein expression (data not shown). Also, there was no induction of VEGF mRNA in these cells by bevacizumab (data not shown).

### Bevacizumab blocks VEGF internalization by ECs

**Figure 5:**
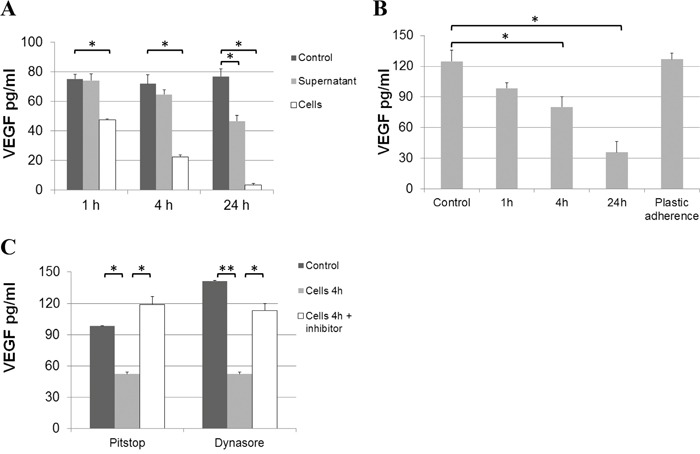
Loss of VEGF from endothelial culture supernatant **A.** 100 pg/ml of hrVEGF-165 were incubated with unconditioned (control), pre-conditioned (supernatant) EC medium or were directly added to confluent EC cultures (cells). The VEGF concentration was determined in the medium by ELISA after 1, 4 and 24 h of incubation. **B.** Furthermore, loss of VEGF was determined for subconfluent (proliferating) EC cultures. The possible effect of protein adherence to plastic was assessed by comparing VEGF concentrations in unconditioned medium without (control) or with (plastic adherence) incubation in an empty culture well for 24 h. **C.** VEGF loss from culture supernatant is due to internalization by ECs. When Pitstop 2 (33 μM) or Dynasore (250 μM) were added to EC cultures supplied with 100 pg/ml hrVEGF, the loss of VEGF from supernatant (after 4 h of incubation) was prevented. (* p<0.05; ** p<0.01).

ECs are known to endocytose VEGF/receptor complexes, partly resulting in complex degradation and partly mediating recycling to the cell surface [31]. It has previously been shown that this internalization process can be blocked by Pitstop 2 or Dynasore [31]. When hrVEGF was added to confluent EC cultures, we detected a gradual loss of VEGF in the supernatant over 24 hours. VEGF decreased by about 30% within 1 h, 70% reduction was detected at 4 h and over 90% of VEGF was lost after 24 h (Figure 5A). The effect was seen for both, confluent and proliferating cells (Figure 5B) and was dependent on the presence of cells, as incubation of hrVEGF with pre-conditioned EC supernatant (in the absence of cells) led to a minor loss of VEGF. Furthermore, VEGF was not reduced by mere plastic adherence to culture wells filled with medium (in the absence of cells, Figure 5B). Importantly, the loss of VEGF in EC supernatant was blocked by the addition of endocytosis inhibitors such as Pitstop 2 or Dynasore further substantiating a mechanism of VEGF internalization and partial degradation by ECs (Figure 5C).

**Figure 6:**
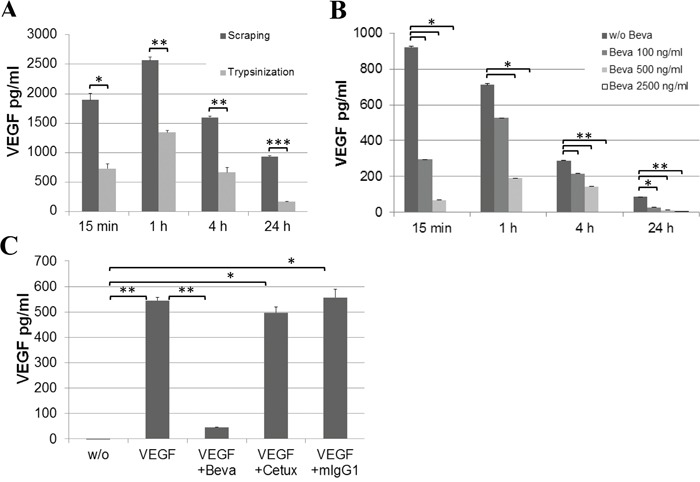
Uptake of VEGF by EC cultures Confluent EC cultures were supplied with 20 ng/ml hrVEGF and cell extracts were prepared at the indicated time points. **A.** Cell harvest by scraping versus trypsinization was compared. **B.** A dose-dependent reduction of VEGF internalization was observed upon addition of bevacizumab at 100, 500 or 2500 ng/ml. **C.** Specificity of inhibition was confirmed by comparing the effect of bevacizumab at the highest concentration (2.5 μg/ml) to cetuximab (2.5 μg/ml) or unrelated mIgG_1_ antibody (50 μg/ml) after 1 h of incubation. (* p<0.05; ** p<0.01; *** p<0.001).

In line, we measured the intracellular VEGF uptake by ECs over time. While cell extracts from untreated ECs were essentially negative for VEGF protein, the addition of hrVEGF to endothelial culture supernatant resulted in a rapid uptake of VEGF within 15 min, with peak levels detected after 1 h by ELISA. Intracellular VEGF levels remained highly elevated after 4 – 24 hours of incubation (Figure 6A). Of note, lower VEGF uptake was measured when cells were collected by trypsinization as opposed to cell scraping, indicating that about 50% of VEGF was bound to the cell surface and hence degraded by trypsin treatment prior to preparation of cell extracts. To detect internalized as opposed to cell-associated VEGF in all subsequent experiments, cells were consistently harvested by trypsinization. The addition of bevacizumab to EC cultures supplied with hrVEGF showed a dose-dependent blockade of VEGF uptake (Figure 6B). The inhibition was specific for bevacizumab, as treatment with cetuximab or mIgG_1_ antibodies had no impact on VEGF uptake (Figure 6C). It should be noted that the inhibition of endothelial VEGF uptake by bevacizumab was only determined in cell extracts but not in cell supernatant, since VEGF detection by ELISA was found to be sensitive to high amounts of bevacizumab in the supernatant but was not impaired in cell extracts - presumably due to lack of efficient bevacizumab internalization (Supplementary Figure S2).

## DISCUSSION

The aim of this translational study was to investigate the effect of anti-VEGF therapy on circulating VEGF levels in a clinical as well as laboratory setting to reveal the mechanism of “VEGF feedback induction” in bevacizumab patients.

In this study we confirmed our previous observation [32] regarding the strong elevation of VEGF levels in plasma of patients with CRC liver metastases after neoadjuvant/conversion therapy with bevacizumab. Importantly, a comparable increase in VEGF blood concentration was observed by both, neoadjuvant and adjuvant treatment. Even though we found that most of the VEGF molecules are antibody-bound and hence inactive, the rise in VEGF could represent a potential escape mechanism and hence be related to treatment response. However, VEGF levels during neoadjuvant therapy showed no association with radiological response which has also been reported in a number of previous studies [33]. In contrast, CEA reduction under neoadjuvant treatment reflected response to therapy, as we have recently observed for a comparable, larger collective of bevacizumab patients [34].

Even though VEGF-bevacizumab complexes would be expected to be rapidly cleared via interaction with Fcγ receptors on blood cells [35, 36], the immunoprecipitation experiments indicated that circulating VEGF accumulates despite being bound by the antibody. It has recently been shown that bevacizumab can indeed form large immune-like complexes with dimeric VEGF-165 resulting in increased affinity for Fcγ receptors [35]. However, VEGF-bevacizumab complexes were also found to exhibit enhanced binding to heparin and neuropilin-1 present on the surface of various cell types. This may in part explain why VEGF-bevacizumab complexes are not readily cleared from circulation by FcR binding but may be diverted to other cell surface molecules. Alternatively, accumulation of complexes may simply exceed the speed of clearance.

The rise of circulating VEGF upon adjuvant administration of bevacizumab suggested that the antibody–triggered increase was independent of the tumor and mostly host-derived. The consistent decrease in CEA levels during neoadjuvant therapy, surgery and adjuvant therapy confirmed the tumor reduction due to treatment and the essential absence of detectable tumor in the adjuvant period. Our findings in the clinical setting are in line with mouse studies which have previously reported that the rise in circulating VEGF after anti-VEGF (receptor) therapy is primarily a host function. “VEGF feedback induction” was observed in non-tumor bearing mice exposed to anti-VEGFR-2 antibody [17] or soluble VEGFR-2 competitor administered by adenoviral gene transfer [21]. With respect to the potential VEGF source, Schmitz *et al*. suggested increased VEGF synthesis by hepatocytes [21] while Bocci *et al*. [17] did not detect elevated VEGF levels in mouse liver. Therefore, we examined the expression of VEGF in resected liver metastases, focusing on both, tumor tissue and surrounding liver. It should be noted that this analysis could only be conducted on tissue from 6 patients which represents a limitation of the current study. The expression of VEGF in CRC cells of resected liver metastases was detectable at the mRNA level and did not correlate with VEGF protein levels measured in plasma. Of note, VEGF expression in tumor tissue was independent of the neoadjuvant regimen with or without bevacizumab. These findings were in accordance with our *in vitro* experiments showing that bevacizumab treatment of two different CRC cell lines did not lead to a specific increase in VEGF expression. Although increased VEGF synthesis by hepatocytes was previously reported for a mouse study on soluble VEGFR-2 therapy [21], the ISH staining showed no substantial VEGF expression in resected liver tissue of our patients, and treatment with bevacizumab did not influence VEGF mRNA levels in hepatocytes. These divergent results might be explained by the fact that an adenoviral vector was employed for drug delivery in the mouse experiment which is known to primarily target the liver and hence might specifically alter hepatocyte biosynthesis.

As platelets represent one of the major sources of VEGF [7], a potential impact of bevacizumab on platelets was also investigated. Despite elevated levels of plasma VEGF, the content of VEGF per platelet remained unchanged. In line, Bocci *et al*. did not detect an impact of anti-VEGFR-2 therapy on platelet-derived VEGF in mice [17]. Since most investigated patients did not experience a change in platelet count, platelet VEGF content or serum VEGF levels, VEGF released by platelet activation did not seem a likely source of increased plasma VEGF after bevacizumab therapy. The ability of platelets to scavenge VEGF has been previously reported [7, 37]. However, our results indicate that, even though exposed to higher levels of plasma VEGF, platelets do not scavenge more VEGF, possibly due to complexation by bevacizumab.

Constituting a central player in angiogenesis, endothelial cells and fibroblasts were also investigated as the potential source of VEGF expression in response to bevacizumab treatment. Endothelial cells are readily stimulated by VEGF and proliferating ECs may produce low amounts of VEGF [38]. However, our *in vitro* experiments detected no substantial VEGF expression in untreated ECs or fibroblasts. The exposure to bevacizumab did not lead to specific increases in VEGF protein expression.

The obtained data triggered the notion that the rise in circulating VEGF during anti-VEGF therapy might be regulated at the level of protein stability and clearance rather than by alterations in gene expression. Hsei *et al*. have previously observed that the systemic clearance of hrVEGF-165 injected into rats was 3-fold reduced when VEGF was pre-complexed with bevacizumab, but they did not further explore the underlying mechanism [27]. It has been reported that sprouting ECs have high rates of VEGF uptake by VEGF receptor endocytosis and VEGF turnover [31, 39]. VEGFR-2 is subject to ligand-activated endocytosis by clathrin- and dynamin-dependent pathways [40]. In our experiments, when hrVEGF was added to EC cultures, we detected a substantial time-dependent loss of VEGF from EC supernatant in both, confluent and proliferating EC cultures. The addition of endocytosis inhibitors (Pitstop 2 or Dynasore) prevented the loss of hrVEGF in supernatant, which indicates that the process was endocytosis-driven. In line, an increase in intracellular VEGF was detectable in endothelial cell extracts. The *in vitro* experiments clearly showed a dose-dependent and antibody-specific blockade of VEGF uptake by bevacizumab. These results led us to the conclusion that bevacizumab binding to VEGF prevents VEGF receptor interaction, and thereby blocks the endocytotic clearance of VEGF by ECs. While we cannot exclude additional mechanisms, this effect is the likely explanation for the accumulation of VEGF in the plasma of patients treated with bevacizumab.

Of interest, a systems biology approach has previously been pursued to compute the potential mechanism of “VEGF feedback induction” by bevacizumab and was based on a whole-body pharmacokinetic model of blood, normal tissue, and tumor tissue [28]. The model predicted a plasma rise in VEGF due to antibody-mediated transport of tissue-derived (interstitial) VEGF into circulation. However, a substantial rise of blood VEGF levels (mostly complexed by bevacizumab) was also predicted when omitting tissue transport from the model, and hence the authors did not exclude another mechanism based on reduced VEGF clearance.

In conclusion, our findings confirm pre-clinical observations of a host (rather than tumor) mediated VEGF increase in response to anti-VEGF therapy. While we could not verify enhanced VEGF synthesis by hepatocytes, platelets or stromal cells, the data substantiate a mechanism based on reduced VEGF clearance from circulation due to decreased VEGF internalization and degradation by endothelial cells. With respect to the clinical relevance, these findings emphasize that circulating VEGF levels in bevacizumab-treated cancer patients are not related to tumor presence and hence do not constitute a mechanism of tumor escape. Furthermore, this may explain why treatment-induced VEGF levels do not predict patient response to therapy.

## MATERIALS AND METHODS

### Study collective of cancer patients

Sixty patients with liver metastases of colorectal cancer, whose primary tumor was resected prior to liver surgery, were included in the study. Patients were divided in two treatment arms receiving neoadjuvant (or conversion) treatment with or without the addition of bevacizumab. Patients were treated with several different chemotherapy regimens: XELOX (oxaliplatin at 85 mg/m^2^ on day 1, capecitabine at 1500 mg/m^2^ twice daily during the first week, followed by 1 week of rest period), FOLFOX6 (oxaliplatin at 100 mg/m^2^, leucovorin at 400 mg/m^2^, a bolus of 400 mg/m^2^ 5′-fluorouracil (5-FU) on day 1 and 2800 mg/m^2^ 5-FU infusion) or FOLFIRI/FOLFOXIRI (irinotecan at 165 mg/m^2^ and 200 mg/m^2^ leucovorin followed by a 400 mg/m^2^ bolus of 5-FU on day 1 and 46 h continuous infusion of 5-FU at 3200 mg/m^2^; in the FOLFOXIRI regimen, oxaliplatin at 85 mg/m^2^ was given additionally on day 1). All treatments were administered bi-weekly for a median of six cycles where bevacizumab was generally omitted from the last cycle. The efficacy of chemotherapy was assessed through the Response Evaluation Criteria in Solid Tumors (RECIST) guideline. After surgical removal of liver metastases a comparable adjuvant regimen was administered.

### Blood collection, plasma and serum preparation

Blood samples were collected from the patients at six different time points: before neoadjuvant treatment (pre-neo), 4-6 weeks after the beginning of neoadjuvant therapy (intra-neo), at the end of neoadjuvant treatment (post-neo), immediately (i.e. 1 day) before operation (pre-OP), before (pre-adj) and after (post-adj) adjuvant therapy.

The investigations were not considered a “clinical trial” but were conducted according to the principles of the Declaration of Helsinki. The Institutional Ethics Committee at the Medical University of Vienna approved the analysis of blood samples (#300/2006, #437/2006, #791/2010); all patients gave written informed consent.

Platelet-free plasma was prepared as previously described [32, 41]. Briefly, blood (10 ml) was drawn into chilled tubes containing CTAD (sodium citrate, theophylline, adenosine, and dipyridamole) as anticoagulants, was kept on ice and further processed within 30 min. After an initial centrifugation step at 1000 x g and 4°C for 10 min, the plasma supernatant was subjected to further centrifugation at 10 000 x g and 4°C for 10 min and stored in aliquots at −70°C. For the preparation of serum, blood samples were collected without the addition of anticoagulants. Blood clotting was allowed to proceed for 1 h before centrifugation at 1000 x g and room temperature (RT) for 10 min.

The levels of VEGF in plasma were determined by ELISA (enzyme-linked immunosorbent assay). The measurement was based on the commercially available Human VEGF ELISA Kit and was performed according to the manufacturer's protocol (Invitrogen Corp., Carlsbad, CA, USA). Serum CEA levels and blood cell count were available from data collected during routine hospital evaluation.

### Immunoprecipitation of VEGF in plasma samples

Removal of human IgG (including bevacizumab) from plasma samples was carried out as we have previously described [19]. 200 μl of plasma were combined with 100 μl of protein A/G PLUS-agarose (Santa Cruz Biotechnology, Inc., Dallas, TX, USA). After 4 h of sample rotation at 4°C and centrifugation for 5 min at 1000 x g, 200 μl of supernatant were again mixed with 100 μl of protein A/G PLUS-agarose and subjected to rotation over night. After two consecutive centrifugation steps, the supernatant was analyzed by ELISA for VEGF content. The established concentrations were multiplied by a factor of 1.6 to adjust for the dilution of samples in the immunoprecipitation (IP) procedure.

### VEGF analysis in platelet extracts

Platelets were isolated from citrated whole blood. Centrifugation at 125 × g for 20 min at room temperature was applied to generate 1 ml of platelet-rich plasma, followed by gel filtration using a sepharose 4B column with HEPES-Tyrode buffer containing 0.5% human serum albumin and 10 μM prostaglandin E1 (Sigma-Aldrich, St. Louis, MO, USA) to obtain a pure and plasma protein – free platelet suspension. After another centrifugation step at 3000 × g for 1.5 min, platelets were lysed in 100 μl Laemmli sample buffer and extracts analyzed for VEGF content by immunoblotting.

### VEGF detection in tumor cell, fibroblast and endothelial cell cultures

Microvessel endothelial cells isolated from human skin [42] were seeded at a density of 5 × 10^5^ in 30 mm wells for 24 h in EGM2-MV growth medium (Clonetics®, Lonza, Walkersville, MD) containing 4 μg/ml fibronectin, 5% fetal calf serum (FCS) and human growth factors without the supplementation of VEGF. The colorectal cancer cell lines HT29 (HTB-38) and SW620 (CCL-227) were originally obtained from ATCC (5/2000), frozen and used in experiments below passage 25. Tumor cells were seeded at a density of 5 × 10^5^ in 30 mm wells for 24 h in McCoy5A and L15 medium respectively, containing 10% FCS, 2 mM L-glutamine and 100 μg/ml penicillin/streptomycin. Fibroblasts isolated from human skin [42] were seeded at 3.5 × 10^5^ in 30 mm wells previously coated with 0.5% gelatin, in MEM medium containing 20% FCS, 2 mM L-glutamine, 1 mM sodium pyruvate, 100 μg/ml gentamycin and 500 ng/ml amphotericin. After 24 h of incubation, cells had generally formed a confluent layer, and were rapidly washed twice (within 2-3 min) with phosphate-buffered saline without Ca^2+^ and Mg^2+^ (PBS) without disturbing or detaching the confluent cell layer. Medium was changed to EGM2-MV with or without 100 pg/ml human recombinant VEGF-165 (hrVEGF) but without supplementation of other growth factors or FCS. After another 24 h of conditioning, the medium was renewed and supplemented with 50 μg/ml bevacizumab. For negative control, cetuximab was applied at the same concentration. Analyses were conducted after 24 or 48 h of cell incubation with antibodies. Cultured cells were washed twice, harvested by trypsinization and disrupted with sodium dodecyl sulfate (SDS) lysis buffer (1% SDS, 100 mM TRIS, pH 9.5) supplemented with protease inhibitors (cOmplete Protease Inhibitor Cocktail Tablets, F. Hoffmann La-Roche AG, Basel, Switzerland). Samples were heated for 5 min at 95°C. Subsequently, sonication was performed for 1 min with the UIS250L Ultrasonic Processor and Sonotrode LTS24d10.4L2 (Hielscher, Teltow, Germany) set to 90% power (amplitude) and 30% sonication (cycle). Samples were kept on ice until centrifugation for 20 min at 14 000 x g and 4°C. The obtained cell extracts were further analyzed for VEGF content by ELISA and immunoblotting.

### Evaluation of VEGF internalization by endothelial cells

Uptake of VEGF was first investigated by loss of VEGF from endothelial culture supernatant. To this end, ECs were seeded in 30 mm wells at a density of 5×10^5^ for confluent and 5×10^4^ for proliferating cultures in endothelial growth medium EGM2-MV containing 4 μg/ml fibronectin, 5% FCS and human growth factors without the supplementation of VEGF. After incubation for 24 h, cells were washed with PBS and 500 μl of EBM2 basal medium were added containing 0.25% bovine serum albumin (BSA) but no growth factors or FCS. The supernatant was collected after 1, 4, and 24 h, briefly centrifuged at 3000 x g for 5 min and separately incubated (cell-free) with 100 pg/ml hrVEGF-165 (PromoKine, Heidelberg, Germany) for the corresponding incubation times of 1, 4 and 24 h to determine VEGF degradation by cell-released factors. Alternatively, 500 μl of EBM2 basal medium were added directly to PBS-washed EC cultures including 0.25% BSA as well as 100 pg/ml of hrVEGF and cultures were incubated for 1, 4 and 24 h to determine loss of hrVEGF from supernatant in the presence of cells. Where indicated, ECs were pre-incubated for 30 min with the endocytosis inhibitors Pitstop 2 (Abcam, Cambridge, UK) and Dynasore (Sigma-Aldrich) at 33 μM and 250 μM respectively, prior to the addition of hrVEGF. Finally, supernatants were collected and analyzed for hrVEGF content by ELISA.

VEGF internalization by ECs was further monitored in cell extracts, i.e. ECs were again seeded in 30 mm wells at a density of 5×10^5^ in EGM2-MV containing 4 μg/ml fibronectin, 5% FCS and human growth factors without the supplementation of VEGF. After 24 h, the confluent cultures were washed with PBS and 500 μl of EBM2 basal medium containing 0.25% BSA and 20 ng/ml of hrVEGF were added. Incubation was performed without and with the addition of bevacizumab at 100, 500 and 2500 ng/ml for 15 min, 1, 4 and 24 h. For negative control, 2500 ng/ml cetuximab or 50 μg/ml of mIgG_1_ (BD Pharmingen, San Jose, CA) were applied. Cultured cells were washed twice, harvested either by scraping or by trypsinization (to degrade extracellular VEGF attached to ECs) and disrupted with SDS lysis buffer supplemented with protease inhibitors. Cell extracts were obtained by sonication (as specified above) and diluted 1:5 with assay diluent buffer for VEGF analysis by ELISA.

Degraded VEGF was not expected to interfere with ELISA detection (in the sense that VEGF fragments might compete for antibody binding and therefore lower the signal for non-degraded VEGF), since the amount of hrVEGF-165 added to culture supernatant (100 pg/ml) was in the lower detection range of the ELISA where antibody availability should not be limiting. Similarly, cell extracts were appropriately diluted prior to ELISA measurement.

### Immunoblotting

Protein concentration of cell extracts was determined using the BCA Protein Assay Kit (Pierce; Thermo Fisher Scientific). Equal amounts of protein (generally 10–25 μg per lane) were resolved by SDS-PAGE and transferred to a polyvinylidene difluoride membrane by semi-dry blotting for 1 h. After blocking with 2% nonfat milk powder in 0.5% Tween 20 in PBS, the membrane was incubated with the primary anti-human VEGF antibody sc-152 (A-20, Santa Cruz Biotechnology, Inc.) in 1:200 dilution at 4°C overnight and subsequently with peroxidase-conjugated secondary antibody sc-2004 (Santa Cruz Biotechnology, Inc.) in 1:5000 dilution for 1 h at RT. Antibody binding was visualized with the SuperSignal West Femto detection system (Pierce; Thermo Fisher Scientific) and the G:BOX imaging system (Syngene, Cambridge, UK).

### *In situ* hybridization

*In situ* hybridization (ISH) for VEGF mRNA was performed as described previously [43]. Briefly, the paraffin-embedded liver tissue sections with colorectal cancer metastases were deparaffinized by three sequential incubation steps with xylol for 20 min at RT, rehydrated to diethylpyrocarbonate-treated water, followed by post-fixation with 4% paraformaldehyde for 20 min. After rinsing with Tris-buffered saline (TBS), slides were incubated with 0.2 N HCl and treated with 20 μg/ml proteinase K for 20 min at 37°C. Digestion was stopped with cold (4°C) TBS. Furthermore, the endogenous alkaline phosphatase (AP) was blocked by a mixture of TBS pH 8.0 and 0.5% acetic acid anhydride. Slides were then dehydrated through graded ethanol and dried with chloroform. Labeling of the anti-sense probe was achieved by *in vitro* transcription for 2 h at 37°C of the linearized plasmid (covering 530 bp of human VEGF-A cDNA) with a nucleotide mix containing digoxigenin-labeled dUTP. For negative control, a sense RNA probe was comparably generated. Slides had to be humidified in a wet chamber for 15 min before applying the hybridization mix containing the digoxigenin-labeled probe in 2x standard saline citrate buffer (SSC), 47% formamide, 10% dextran sulfate, 0.01% sheared DNA and 0.02% SDS. Tissues were protected with a cover slip and incubated in the humidified chamber at 65°C overnight. After hybridization, slides were washed vigorously three times with 50% formamide in 1x SSC for 20 min at 55°C, followed by rinsing twice for 15 min in 1x SSC at RT. Visualization steps were performed by blocking with Roche blocking reagent (F. Hoffmann La-Roche AG, Basel, Switzerland) for 15 min and applying anti-digoxigenin antibody – AP conjugate 1:500 (Roche) in blocking reagent for 1 h at RT. Finally, slides were immersed in nitro-blue tetrazolium and 5-bromo-4-chloro-3-indolyphosphate substrate solution and developed overnight. Images were taken with a Polyvar 2 microscope (Reichert, Vienna, Austria).

### Statistical analysis

With respect to the investigated number of patients, group size calculations were conducted with the tool available at http://powerandsamplesize.com/Calculators/Compare-2-Means/2-Sample-Equality and were relying on VEGF plasma values previously measured in individuals after neoadjuvant chemotherapy with or without bevacizumab [19]. The calculated sample size based on a power of 0.90 and alpha error of 5%, was N=21 (bevacizumab-treated patients) and N=7 (chemotherapy controls) for the neoadjuvant setting and a sampling ratio of 3:1. Comparably, the calculated group sizes were N=26 and N=6 for the adjuvant setting with a sampling ratio of 4.3:1. Hence, the numbers of investigated patients were matching (adjuvant) or exceeding (neoadjuvant) the required sample size.

The analysis of acquired data was based on non-parametric tests, using SPSS software version 21 (SPSS Inc., Chicago, IL, USA). Therapeutic changes of circulating markers and comparison of samples with and without VEGF immunoprecipitation were determined by Wilcoxon test. The Mann-Whitney-U test was applied to assess differences between treatment arms. Statistical analyses of *in vitro* experiments were conducted with unpaired T-test. P-values < 0.05 were considered statistically significant.

## SUPPLEMENTARY MATERIALS FIGURES


