# Supplementary material for: The VEGF rise in blood of bevacizumab patients is not based on tumor escape but a host-blockade of VEGF clearance
Source: Oncotarget. 2016 Aug 5;7(35):57197–212. doi: 10.18632/oncotarget.11084 (PMC5302983; doi:10.18632/oncotarget.11084)
Supplement: Supplementary file 1 [file oncotarget-07-57197-s001.pdf]

# The VEGF rise in blood of bevacizumab patients is not based on tumor escape but a host-blockade of VEGF clearance

## SUPPLEMENTARY FIGURES

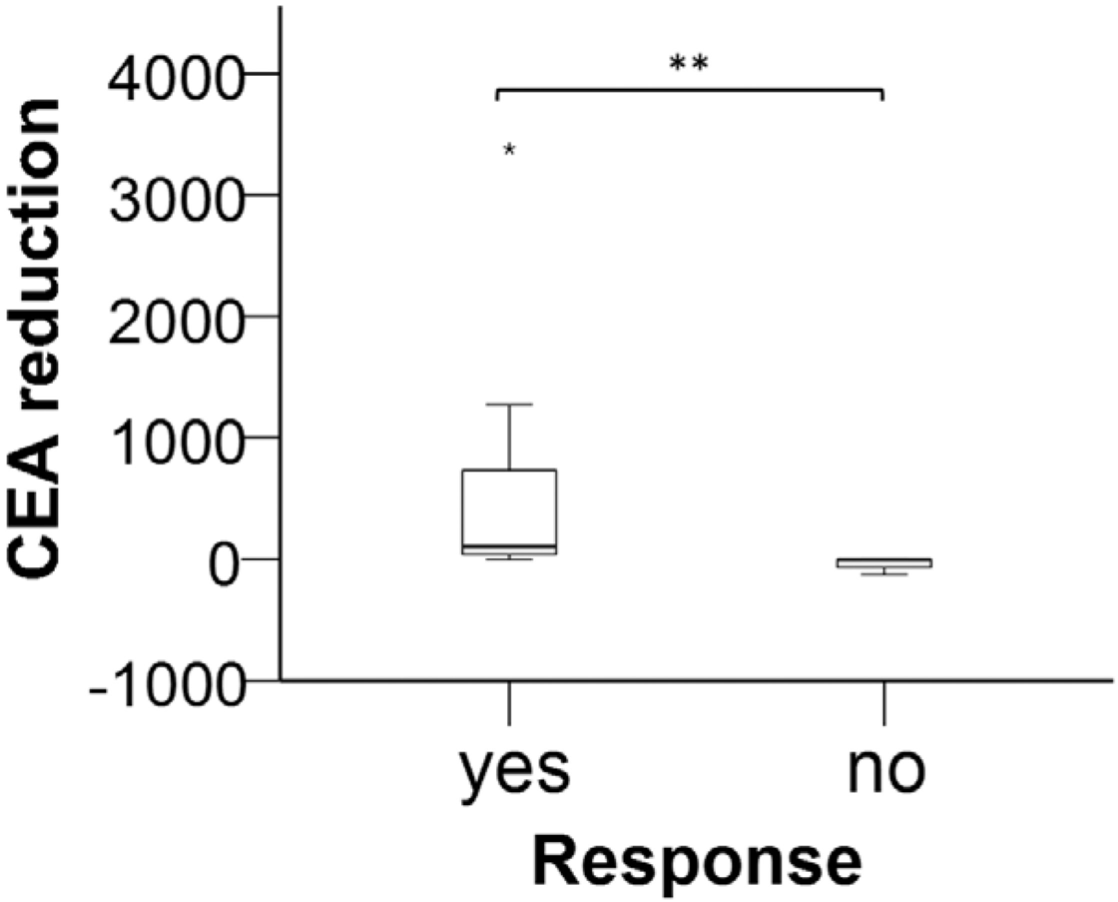


**Supplementary Figure S1: Association of tumor marker CEA with radiological response (RECIST) of patients neoadjuvantly treated with chemotherapy and bevacizumab.** Changes in CEA levels (µg/l) were monitored if elevated before treatment. The reduction in CEA levels during neoadjuvant therapy (post- versus pre-neo) differed significantly between patients with (CR or PR) or without (SD or PD) response to treatment. (** $p<0.01$).

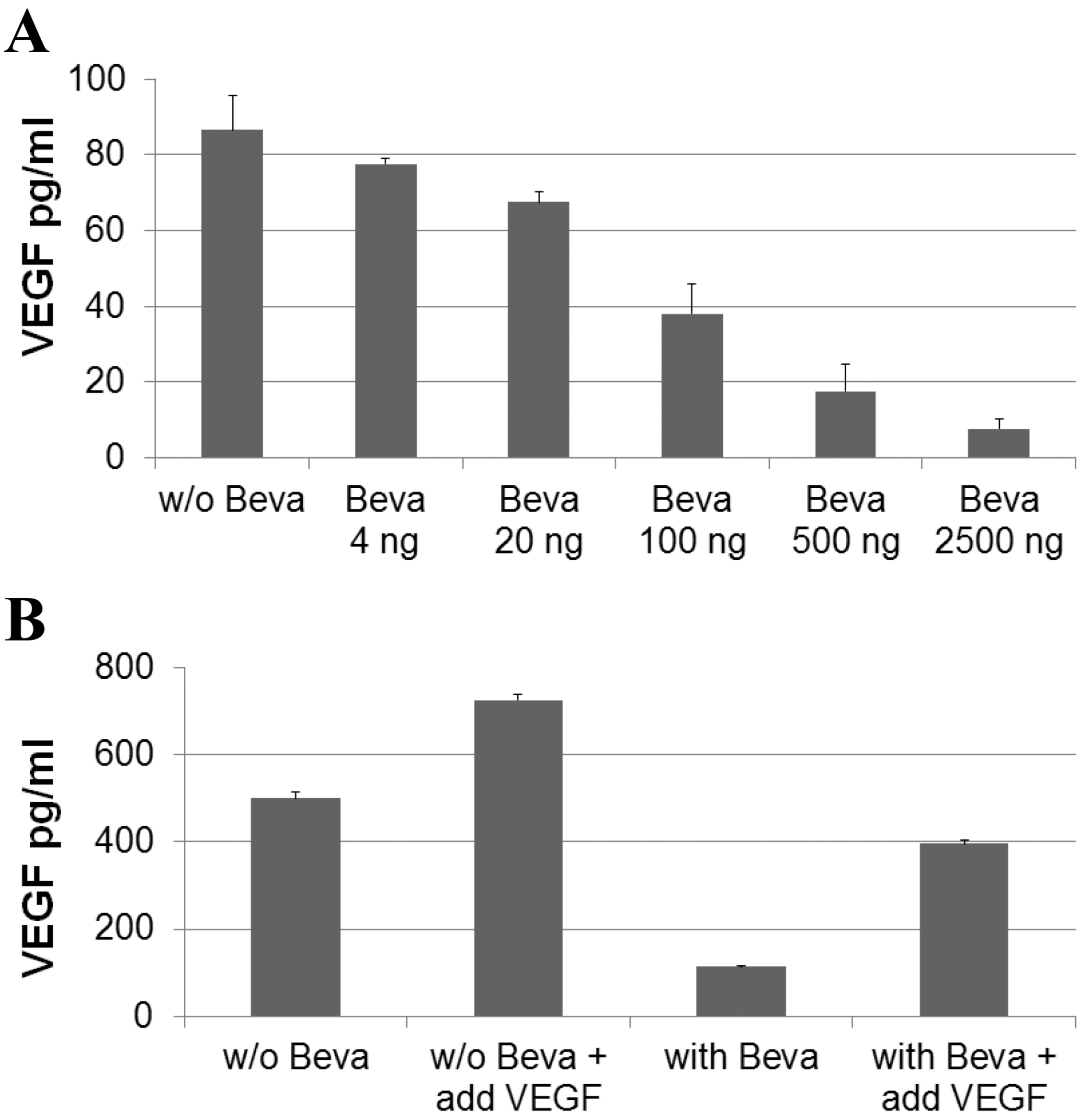


**Supplementary Figure S2: Interference of bevacizumab with VEGF ELISA measurements. A.** The influence of bevacizumab was determined when added directly to basal EBM2 medium with 0.25% BSA containing 100 pg/ml of hrVEGF. Bevacizumab was titrated at 4, 20, 100, 500 and 2500 ng/ml. **B.** When EC cultures were supplied with 20 ng/ml of hrVEGF with or without the addition of 1000 ng/ml of bevacizumab for 1 h, cells were subsequently harvested by trypsinization and extracts were tested by ELISA for hrVEGF content. To establish whether bevacizumab might be present in cell extracts and interfere with VEGF detection by ELISA, cell extracts were further mixed with an additional 300 pg/ml of hrVEGF (add VEGF). While bevacizumab blocked uptake of VEGF into endothelial cells, cell extracts did not seem to contain free/functional antibody, as the increase by 300 pg/ml added VEGF was readily detectable.
